# A Sensor Array Using Multi-functional Field-effect Transistors with Ultrahigh Sensitivity and Precision for Bio-monitoring

**DOI:** 10.1038/srep12705

**Published:** 2015-07-30

**Authors:** Do-Il Kim, Tran Quang Trung, Byeong-Ung Hwang, Jin-Su Kim, Sanghun Jeon, Jihyun Bae, Jong-Jin Park, Nae-Eung Lee

**Affiliations:** 1 School of Advanced Materials Science & Engineering, Sungkyunkwan University, Suwon, Kyunggi-do 440-746, Republic of Korea; 2 SKKU Advanced Institute of Nano Technology (SAINT), Sungkyunkwan University, Suwon, Kyunggi-do 440-746, Republic of Korea; 3 Department of Applied Physics, Korea University, Sejongro 2511, Sejong 339-700, Korea; 4 Samsung Advanced Institute of Technology, Samsung Electronics Corporation, Yongin, Kyunggi-do 446-712, Republic of Korea; 5 School of Polymer Science & Engineering, Chonnam National University, Gwangju 500-757, Korea

## Abstract

Mechanically adaptive electronic skins (*e*-skins) emulate tactition and thermoception by cutaneous mechanoreceptors and thermoreceptors in human skin, respectively. When exposed to multiple stimuli including mechanical and thermal stimuli, discerning and quantifying precise sensing signals from sensors embedded in *e*-skins are critical. In addition, different detection modes for mechanical stimuli, rapidly adapting (RA) and slowly adapting (SA) mechanoreceptors in human skin are simultaneously required. Herein, we demonstrate the fabrication of a highly sensitive, pressure-responsive organic field-effect transistor (OFET) array enabling both RA- and SA- mode detection by adopting easily deformable, mechano-electrically coupled, microstructured ferroelectric gate dielectrics and an organic semiconductor channel. We also demonstrate that the OFET array can separate out thermal stimuli for thermoreception during quantification of SA-type static pressure, by decoupling the input signals of pressure and temperature. Specifically, we adopt piezoelectric-pyroelectric coupling of highly crystalline, microstructured poly(vinylidene fluoride-trifluoroethylene) gate dielectric in OFETs with stimuli to allow monitoring of RA- and SA-mode responses to dynamic and static forcing conditions, respectively. This approach enables us to apply the sensor array to *e*-skins for bio-monitoring of humans and robotics.

Next-generation applications of flexible electronics have been recently demonstrated such as transistors^1^,^2^,^3^,^4^,^5^, wearable devices^6^,^7^,^8^,^9^,^10^,^11^,^12^, skin-attachable sensors^13^,^14^,^15^,^16^,^17^,^18^,^19^, and electronic skin^13^,^14^,^15^,^16^,^17^,^18^,^19^,^20^,^21^,^22^,^23^,^24^,^25^,^26^,^27^,^28^,^29^,^30^,^31^,^32^,^33^,^34^,^35^ (*e*-skin). Among those, flexible *e*-skins with an array of sensor pixels have many applications including biomedical health monitoring^13^,^14^ and robotics^20^,^21^. An *e*-skin is comprised of arrays of pixels that function as sensing devices for various targeted external stimuli. The different kinds of sensors in a pixel in the *e*-skin emulate sensory receptors in human skin. Sensory receptions in human skin where sensory receptors in neurons are responsive to various physical stimuli include mechanoreception, thermoception and nociception. Recently, there have been extensive studies on *e*-skins that mimic human sensory reception, in particular, of mechanoreception^13^,^18^,^19^,^20^,^21^,^22^,^23^,^24^,^25^, thermoreception^16^,^28^,^35^, or both^26^,^30^,^32^.

In human skin, there are two modes of mechanoreception, rapidly adapting (RA) and slowly adapting (SA) reception. In RA-type reception, the perception of slip or touch is achieved under dynamic pressure. In SA-type 1 (SA1) and SA-type 2 (SA2) reception, the perceptions of form and roughness on the skin and skin stretch (i.e. pressure or strain) under static stimuli, respectively, are achieved. In many previously reported works, *e*-skins in which sensors measure only one sensing parameter in dynamic^31^,^34^ and static mechanical stimuli^14^,^22^ have been investigated. In practical applications of highly functional *e*-skin, e.g. artificial fingers, both RA- and SA-mode detection capabilities of dynamic and static pressure need to be demonstrated. Furthermore, multimodality of *e*-skin, including thermoreception as well as mechanoreception, is also required. However, simultaneous detection of sensitive RA- and SA-mode pressure and temperature in *e*-skin has rarely been demonstrated. Furthermore, quantification of measured signals for precise sensing of arbitrary input stimuli, e.g. pressure and temperature, from the sensing devices in single^13^,^14^ or multimodal^26^,^30^,^34^ *e*-skin have rarely been reported even though their electrical responses to known input stimuli were often measured.

In our previous report on multimodal sensing of *e*-skin^32^, bimodal sensing of pressure and temperature using a single, functional organic field-effect transistor (OFET) with a signal decoupling approach was demonstrated. This approach is unlike a pixel in which multiple sensors are responsive to different stimuli^13^,^14^. In flexible *e*-skin, the target signals from sensing elements under multiple stimuli are often influenced by the mechanical strain experienced by the *e*-skin. We solved the subjected interferences and achieved bimodal sensing of temperature and pressure (or strain) by using an alternating current (AC) gate bias technique^24^,^32^ in an OFET sensor platform directly integrated with both the piezo-pyroelectric gate dielectric and piezo-thermoresistive organic semiconductor channel. However, the lowest detection limit for pressure (50 kPa) in the previous work was relatively high compared to the mechanoreception level of human skin (<10 kPa) due to a limited degree of mechano-electrical coupling of the thin-film gate dielectric and channel in OFETs. Thus, the reported sensing device was not suitable for exploring biomedical health monitoring using *e*-skins even though it possessed the merit of multimodal function. Moreover, RA-type detection of dynamic pressure was not studied in detail.

Herein, we improve our methods to further elaborate the precise detection of unknown, applied minute stimuli of both dynamic and static pressures by measuring the output signals from the OFET platform with highly responsive, microstructured functional gate dielectrics. We further demonstrate the applicability to quantitative bimodal sensing of static pressure and temperature from the microstructured OFET array fabricated on a flexible substrate. Microstructuring of a functional gate dielectric, a highly crystalline, organic ferroelectric material, poly(vinylidenefluoride-trifluoroethylene) (P(VDF-TrFE)), greatly improved the pressure responsivity of the OFET sensor due to larger flexoelectricity-enhanced piezoelectric effects in pyramidal shaped microstructures; pressure as low as 20 Pa was detectible. The sensors could be applied to precise detection of heart beat rate and skin temperature.

## Results

### Device Fabrication

**Figure 1:**
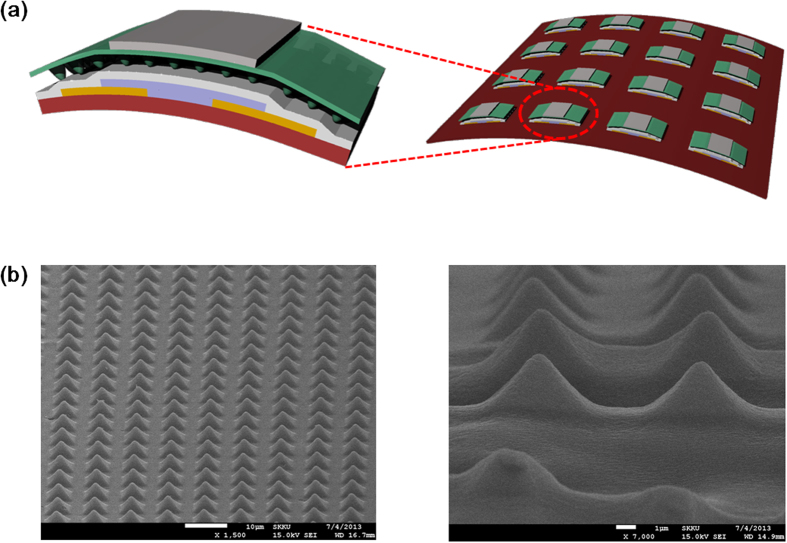
(**a**) The structure of an OFET and a transistor array, comprised of microstructured P(VDF-TrFE) as a gate dielectric and pentacene as a channel layer. (**b**) FE-SEM images of microstructured P(VDF-TrFE) crystallized at 140 °C for 4 hours.

For flexible physical sensors highly responsive to dynamic and static pressures in *e*-skin, we constructed a single OFET sensor or an array of OFET sensors on top of a flexible substrate. We first incorporated a highly crystalline P(VDF-TrFE) as the gate dielectric and an organic semiconductor, pentacene, as the channel to the physically responsive FET (physi-FET) platform (Fig. 1a). Top-gated, bottom-contact OFETs were fabricated on a polyimide (PI) substrate. The procedure for device fabrication is shown in Supporting Fig. S1. Briefly, the source-drain contact formation of the Au layer and sequential deposition of the pentacene layer were carried out. For improved stability of device characteristics, the surface of the pentacene channel was encapsulated by an organic layer of tetratetracontane (TTC).

Highly crystalline, microstructured P(VDF-TrFE) with a pyramidal shape (Fig. 1b) was replicated by using a mould of polyurethane acrylate (PUA) fabricated on polyethylene terephthalate (PET) substrate, crystallized at 140 °C for 4 hrs, subsequently cooled to room temperature, and peeled off from the mould. Here, crystallinity of a thick P(VDF-TrFE) microstructure was observed in the FE-SEM image of the top view (See Supporting Fig. S2). As shown in Supporting Fig. S2, the film showed the best crystallinity after crystallization for 4 hours. Prior to spin coating P(VDF-TrFE), an amorphous-carbon (a-carbon) film was formed on the top of a PUA mould to reduce surface energy, which enabled the P(VDF-TrFE) microstructure to be detached easily from the mould. PUA moulds were fabricated by replication casting using the silicon master mould. Average width, distance and height of pyramids in the microstructured P(VDF-TrFE) gate dielectric were 4, 4, and 2.5 μm, respectively. The 4-μm-thick microstructured P(VDF-TrFE) layer was transferred to the top of the encapsulated pentacene channel and the gate electrode layer was deposited in sequence (see Methods for details). As shown in Supporting Fig. S3, output characteristics of microstructured OFETs indicated slightly high off-current before poling, which can be attributed to the repeatedly applied gate voltage during measuring devices causing a slight poling of the gate dielectric. After poling, devices indicated linear output characteristics due to an internal field induced by polarization of dipoles in the highly crystalline, microstructured P(VDF-TrFE) gate dielectric. An array of 4 × 4 microstructured OFETs was also fabricated using the same process sequence for real-time measurements of sensing parameters by direct addressing of the individual device (Fig. 1a).

### Bimodal detection of temperature and SA-mode pressure

In order to realize SA-mode response characteristics of fabricated OFETs in response to a static and varying pressurizing condition, a cyclic forcing system with a load cell on a metal block heated at different temperatures was used to apply pressure and temperature simultaneously to the device. For precise quantification of SA-mode detection of static pressure, the AC gate bias technique and signal deconvolution method were adopted (as an example, see the sensor responses under AC gate biasing in Supporting Fig. S4)^24^,^32^. Figure 1b shows the responses of channel transconductance, *g*_*m*_, and equivalent voltage, *V*_*0*_, when varying both pressure, *P,* and temperature, *T*, as extracted from an OFET. The observed linearity in modulation of *g*_*m*_ and *V*_*0*_ in Fig. 1b enables us to measure unknown static *P* and *T* inputs. Figure 1c shows the readout values of *T* and *P* (open circles) under stimuli of *T* and *P* from commercial gauges (cross point of dotted lines). The readout values of *T* and *P* from the sensor matched well with the input *T* and *P* parameters. The detectible pressure range of the OFET with microstructured functional gate dielectric for static pressure was much lower than that of the OFET with a thin-film functional gate dielectric. When comparing M values with different gate dielectric structures, M_1_ and M_3_ values when using microstructured P(VDF-TrFE) were much higher than when using the thin-film structure (Supporting Table S1). It is believed that microstructured P(VDF-TrFE) leads to not only a large thickness modulation resulting in an increase in gate dielectric capacitance, *C*, and, in turn, in *g*_*m*_, but also enhanced *V*_*0*_ due to a flexoelectricity-induced piezoelectric effect of pyramidal gate dielectric when pressurized.

### High precision for detecting temperature and SA-mode pressure

**Figure 2:**
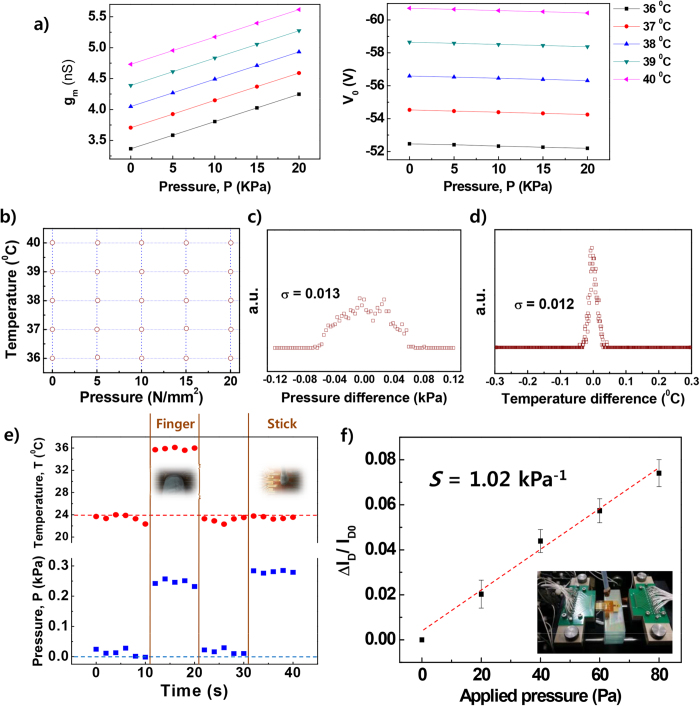
(**a**) The changes of channel transconductance (g_m_) (left) and equivalent voltage (V_0_) (right) in a multi-functional OFET under simultaneously applied pressure (P) from 0 to 20 kPa and temperature (T) from 36 to 40 °C. (**b**) Extracted read-out P and T values (open circles) measured 10 times under stimuli of P and T from commercial gauges (cross point of dotted lines). (**c**) P and (**d**) T differences calculated by 1,000 collected data points from four devices. (**e**) Demonstration of real-time bimodal sensing with stimuli applied by a finger and a blunt stick. (**f**) Change of I_D_ measured at very low pressure range from 0 to 80 Pa with error bars after repeatedly conducted up to 5 times.

In order to investigate temperature and SA-mode pressure detection accuracy of microstructured OFETs, pressure and temperature differences are calculated using Equation (4) (see Fig. 2c,d). Here, four devices were measured 10 times at five different applied *T* and *P* values. From those measurements, 1,000 data points at each *T* and *P* were explored to estimate the statistical accuracy quantitatively. As shown in Fig. 2c, a very small value of the standard deviation in the extracted *P* values (0.013 kPa) was found. We also achieved a very small standard deviation (12 mK) for the extracted *T* values, as shown in Fig. 2d. Very high precision and repeatability of sensing devices are critical for bio-monitoring applications. For example, resolution of skin temperatures <50 mK is essentially required for biomedical monitoring of skin temperature^16^. Indeed, the sensing devices presented in this work can effectively provide precise measurements of skin temperature and heart beat rate.

### Demonstration for bimodal detection of temperature and SA-mode pressure

To demonstrate SA-mode detection of simultaneously applied P and T, the OFETs were put under pressure by a finger and a blunt stick. As shown in Fig. 2e, each data point could be extracted by measuring for only 2 s because I_D_ measured with the AC gate bias was stabilized within 2 s after pressurizing (Supporting Fig. S4). When the sensor was pressed by a finger with a relatively constant pressure (Fig. 2e), the device responded to the finger pressure with a reading of 0.25 kPa. When a thumb was pressed on the sensor, the measured temperature ranged from 35 to 36 °C (Fig. 2e). Considering that skin temperature of human hand varies from 21 to 37 °C depending on subjects and environments, a more detailed study is required for a precise measurement of the different parts of human skin under different conditions^16^,^33^,^36^,^37^,^38^,^39^,^40^,^41^,^42^,^43^. In order to confirm reasonability of our results, therefore, more experiments for investigation of finger temperatures of multiple subjects were carried out by using an IR camera at different ambient conditions (indoors vs. outdoors) after calibration using a thermocouple embedded in the heated metal block that was also used for the calibration of the sensor. As shown in Supporting Table S2, 3 and Supporting Fig. S5, there was a fluctuation (from 33 to 36 °C) in the measured temperatures of human’s thumb from different subjects or under different environments (indoors vs. outdoors). The indoor temperature of the laboratory during IR thermograpy measurements for all the subjects was the same as that during experiments for the data in Fig. 2e. The thumb temperature measured from the subject #1 by the sensor (Fig. 2e) and by the IR camera (Supporting Table S2, 3 and Supporting Fig. S5) was almost the same. Average values of thumb’s temperatures indoors and outdoors were calculated as 21.2 and 34.6 °C, respectively. Based on the results of our and other group‘s estimations for human body temperatures, it is considered that the electrically measured finger temperature in this work is reasonable. Pressed by a blunt tip (Fig. 2e), the pressure was measured as approximately 0.3 kPa but no change in temperature was measured. This demonstration indicates the usefulness of the device to quantitatively measure unknown temperature and static pressure simultaneously. The decoupling of static pressure and temperature enables us to achieve bimodal sensing from a single sensing device.

### Limit of detection and sensitivity in SA-mode pressure detection

In order to investigate the pressure detection limit of the microstructured OFETs, changes in I_D_ were measured while light objects composed of clay were placed on the top of the devices, which was repeatedly performed up to 5 times and the averaged values with a standard deviation were indicated in the Fig. 2f. In the preparation of the measurement system, a vacuum pump was used to hold and drop an object on the surface of OFETs (see the Fig. 2f). Prior to dropping the object, we measured the weight and surface area of a piece of clay to calculate the applied pressure. The microstructured OFET showed superior responses and linearity with a pressure sensitivity of 1.016 kPa^−1^ under a pressure range of 20 ~ 80 Pa when applied by light objects. Thus, the lowest detectible pressure was estimated as 20 Pa, which is comparable to that of a recently reported pressure sensing device^14^. Moreover, we also investigated sensing performance after a cyclic bending test. As shown in Supporting Fig. S6, pressure sensitivity was slightly degraded from 1.016 to 1.013 kPa^−1^, which was caused by degradation of the pentacene channel. The off-state current of the microstructure OFETs was slightly decreased after cyclic deformation (not shown). However, this issue might be resolved if the channel layer were replaced with more stable organic semiconductor materials.

### RA-mode detection of dynamic pressure

**Figure 3:**
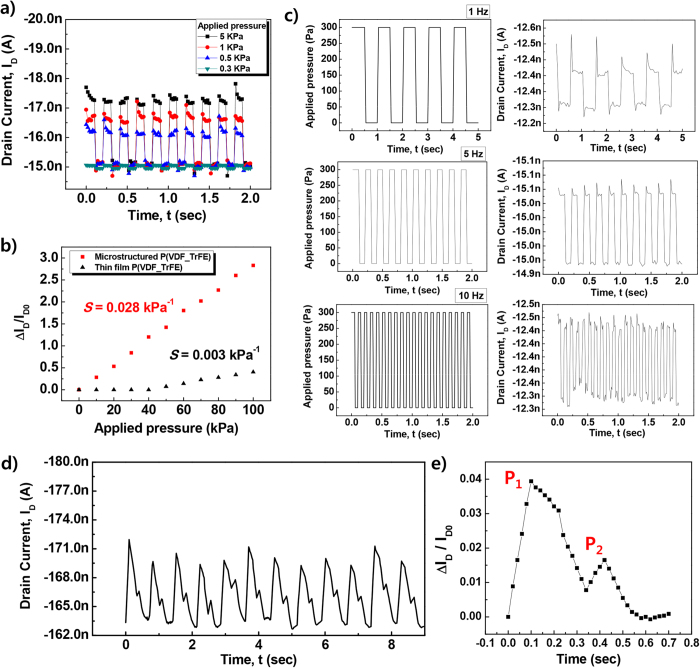
(**a**) The time-dependent I_D_ of multi-functional OFETs with varying pressure from 0.3 to 5 kPa at a frequency of 5 Hz. (**b**) Comparison of sensitivity between OFETs with microstructured and thin-film P(VDF-TrFE) as the gate dielectric. (**c**) Response characteristics (I_D_) of OFET sensors when varying the frequency of applied pressure from 1 to 10 Hz (from the top to the bottom figure) at a fixed pressure of 0.3 kPa. (d) The I_D_ response to a human pulse. (**e**) Enlarged output signals for detecting a human pulse.

In order to understand the RA-mode pressure responsivity of the microstructured OFET sensor, the drain current (I_D_) modulation of microstructured OFET to dynamic pressurizing condition (“apply and release” type) with varying applied force was first measured in a dynamic loading system similar to the system used by Takei *et al*.^31^. Small dynamic pressure ranged from 0.3 to 5 kPa at a forcing frequency of 5 Hz and forcing time of 100 ms. The results are shown in Fig. 3a. In the cyclic forcing test of microstructured OFETs, the lowest applicable pressure was 0.3 kPa due to the limit of the equipment used in the measurements. However, we already demonstrated responsivity to dynamic pressure of microstructured OFETs at a range as low as 20 Pa by dropping light objects (Fig. 2f). The results of the cyclic forcing test indicated highly responsive behaviour of the microstructured OFET to very small P, as low as 0.3 kPa, at a forcing frequency of 5 Hz.

Significant enhancement in responsivity of microstructured OFET sensors to dynamic pressure is primarily attributed to enhanced electro-mechanical coupling effects induced by larger deformation of microstructured P(VDF-TrFE) compared to that of thin-film P(VDF-TrFE) under the same pressurizing condition. Increased deformation of a microstructured gate dielectric can lead to aggravated changes in *C* and *P*_*r*_ in the functional gate dielectric and, in turn, enhanced modulation of I_D_ in the microstructured OFET sensor. Analysis of *g*_*m*_ and *V*_*0*_ changes in the microstructured gate dielectric and responsive characteristics of microstructured OFETs under static pressurizing conditions, as already discussed in SA-mode responses, indicated that a large change in *C*, rather than in *P*_*r*_, of the microstructured gate dielectrics of the OFET is primarily responsible for enhanced modulation of *g*_*m*_ and, in turn, I_D_. In order to determine the causes of creating superior responsivity in RA mode, we have explored the sensitivity of a microstructured OFET compared to the planar-structured OFET over a high pressure range. As shown in Fig. 3b, the change in I_D_ was extracted by increasing the applied pressure by a range from 10 to 100 kPa. As expected, sensitivity of a microstructured OFET (0.028 kPa^−1^) was much higher than that of a planar-structured OFET (0.003 kPa^−1^).

To investigate dynamic responses at varying forcing frequencies, response characteristics of microstructured OFETs subject to low levels of dynamic pressure at 0.3 kPa were measured at a forcing frequency from 1 to 10 Hz. Forcing frequencies larger than 10 Hz could not be applied in this experiment due to the limit of the dynamic forcing equipment. The results in Fig. 3c indicate that at up to 10 Hz of forcing condition, dynamic responses followed the applied forcing frequency without a significant time delay. Response time after dynamic pressurizing is on the order of 20 ms at 10 Hz forcing frequency. This response time is sufficient for tactile sensing applications. The functional gate dielectric is not the limiting factor to realize high-speed tactile sensors as the P(VDF-TrFE) was reported to tolerate and respond up to a very high frequency, above the kHz range^32^. Dynamic responses of the microstructured OFETs at higher forcing frequencies are expected to be limited by low field-effect channel mobility in pentacene OFETs.

### Detection of pulse rate

To demonstrate the applicability of RA-type responses using microstructured OFETs, the devices were applied to detect heart beat rate by sensing pressure modulation in the human radial artery at the wrist. As shown in Fig. 3d, I_D_ of the microstructured OFETs was monitored while the devices were attached to the wrist. The pressure wave of the radial artery typically includes three different waves: an incident wave of blood flow and two reflection waves^44^. However, for young healthy people (below 30 years old) two clearly distinguishable waves are observed^44^; an incident wave and a reflected wave, which is also demonstrate in our results measured from a 27 year old person. For arterial stiffness diagnosis, the radial artery augmentation index (AI_r_ = P_2_/P_1_) is commonly explored by extracting P_1_ and P_2_ in Fig. 3e. The average value of AI_r_ was estimated as 42%, which matches reasonably with data from the literature regarding a healthy adult male in his mid-twenties^44^. Hence, the microstructured OFETs showed superior applicability for a biomedical health monitoring device when measuring heart beat rate.

### Array demonstration of SA- and RA-mode detection

**Figure 4:**
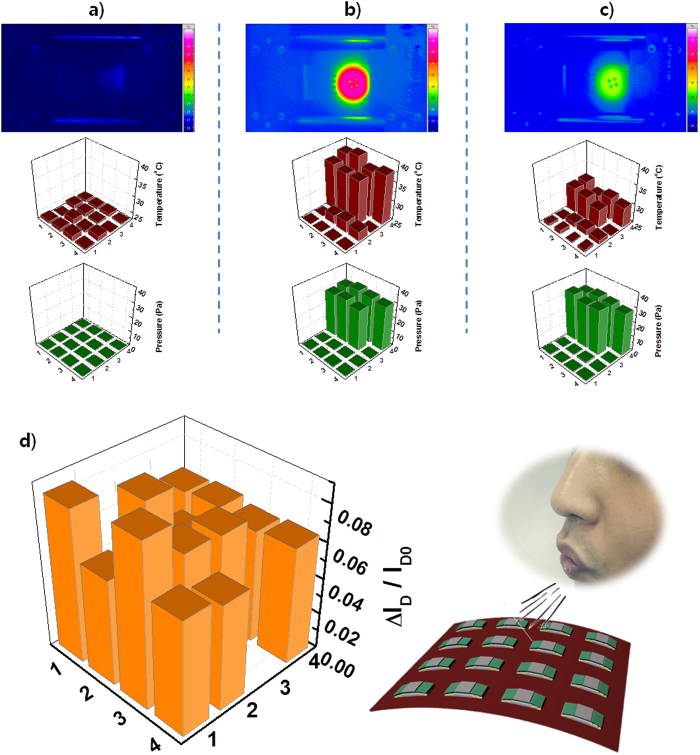
Read-out P and T from the 4 × 4 device array: (**a**) without stimuli and (**b**) with an object heated to up 40 °C placed onto the device array and (**c**) after cooling down for 3 min. (**d**) Change in I_D_ of a sensor array while strongly blowing breath directly onto it. Two devices at (3, 4) and (2, 1) are defective.

To apply the described RA- and SA-mode sensing capability of a microstructured OFET to an array format, real-time RA- and SA-mode sensing of a 4 × 4 device array was demonstrated by measuring real-time responses of the device array. The pixel size containing an OFET was approximately 0.5 × 1 mm, and our 16 devices were uniformly distributed over a 1.3 × 1.3 cm^2^ area. Our array system can simultaneously measure the arbitrary *T*-*P* values of the arrayed 16 devices in real-time by directly addressing each device in the array. The measurement details of the array system were described in the Methods section. Figure 4b shows a three-dimensional mapping of measured *T* and *P*, respectively, for the SA-mode demonstration, when half of the devices, i.e. 8 devices, were pressed by placing a heated button, as shown in the thermal image obtained by an infrared (IR) camera. It was clear that the read-out temperature and pressure of the devices with the heated object were higher than those of the devices without it (see Fig. 4a). Moreover, the read-out temperatures of the devices under the object were also measured to be very close to that of the as-heated temperature (40 °C) obtained from the thermal image. After 3 min, read-out temperatures obtained from the object were decreased due to atmospheric cooling with time (See Fig. 4c). Even though the read-out temperatures of the devices decreased, read-out pressures were not changed due to the same pressurization by the placed object.

To demonstrate RA-mode detection, changes in I_D_ of 16 devices in an array were extracted while breath from the mouth was blown out onto the devices. The distance between the device array and mouth during blowing was 10 cm. As shown in Fig. 4d, devices responded well to the stimuli caused by blowing the breath directly on the surface of the arrayed devices. The results indicate the ultrahigh responsivity of the devices in RA-mode detection. Demonstrations for detection of various dynamic stimuli indicate the potential applicability of the sensors in many different applications in smart devices.

### Precise measurements of temperature distribution of skin

**Figure 5:**
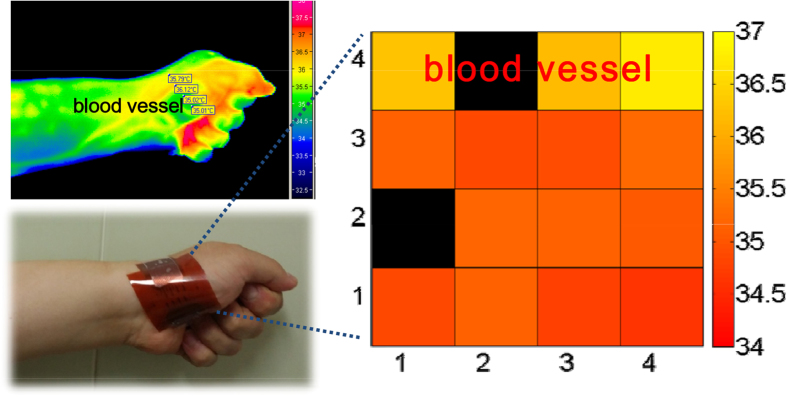
Mapping of the temperature distribution from the 4 × 4 device array onto human skin including the area with a blood vessel and comparing it with the thermal image obtained by an IR camera. The black colour in the temperature distribution map indicates the defective devices.

In order to investigate the applicability of a sensor array for bio-monitoring, we monitored the temperature distribution of the human body after attaching the sensor array onto the human skin. The skin attached device array was slightly bent due to the non-flat human skin area. To realize mechanical durability, therefore, we evaluated the temperature detecting ability of microstructure OFETs under mechanical bending at bending radius of 1 cm. As shown in Supporting Fig. S7, there is almost no difference between applied and read-out temperatures even at the bent state. Therefore, it is considered that bending effect on temperature read-out of the sensor array attached on the palm is negligible. The thermal image of the same area in the skin was also obtained with an IR camera (see the Fig. 5). The read-out temperatures of 16 devices are very close to the skin temperature from the thermal image taken by the IR camera, as shown in Fig. 4e. Furthermore, we discovered that there is a slight difference in the read-out temperatures depending on device position. In the IR image of the human skin, it was clear that the temperature of the blood vessel is slightly higher compared to the temperature at other positions, which was also seen in the read-out temperatures measured from the device array. The superior temperature resolution of our sensor array enabled us to distinguish the temperature of a blood vessel.

## Discussion

We successfully demonstrated *e*-skin comprised of an OFET array using a microstructured P(VDF-TrFE) gate dielectric layer, which can be used to extract the effects from temperature as well as pressure changes in both slowly and rapidly adapting modes. Especially, the microstructured functional gate dielectrics enable the *e*-skin to possess high pressure sensitivity (1.02 kPa^−1^) which is induced by large changes in channel transconductance (*g*_*m*_) caused by a large change in the capacitance (*C*) of the microstructured gate dielectric incorporated into the OFET platform, with a superior linearity and response time of 20 ms in RA-mode pressure detection. In SA-mode multi-stimuli detection of P and T, on the other hand, the developed *e*-skin can quantitatively measure multiple input values with extremely high accuracy within 2 sec even under simultaneously applied pressure and temperature. The *e*-skin, composed of microstructured OFETs, uses an organic semiconductor channel to create mechanical flexibility and ease fabrication compared to inorganic semiconductor materials. Furthermore, various merits of our *e*-skin like superior sensitivity, linearity, flexibility and accuracy enable for it to be applied to bio-monitoring of heart beat rate and skin temperature distribution.

The power consumption is at the level of 10 μW due to the low channel current level of nA at a relatively high operation voltage. However, the low current level caused by high resistance of the channel in our OFETs may result in the low operation speed in the array. The observed high channel resistance is attributed to intrinsically limited carrier mobility of pentacene. On the other hand, the high operation voltage range of our OFET sensor caused by the small capacitance of microstructured gate dielectric with an air gap can be a problem for wearable applications. Further reduction in the power consumption of the sensor with a higher output current and lower operation voltage may be enabled by designing the microstructured gate dielectric with smaller air gap and adopting the channel materials with superior mobility. We anticipate that the *e*-skin of a microstructured, multi-functional OFET array may be useful for next generation smart electronic systems such as wearable electronics, human-machine interfaces or robotics.

## Methods

### Preparation of materials and devices

The P(VDF-TrFE) (65 mol% of VDF) was purchased from Piezotech S.A. (France) Highly crystalline microstructured P(VDF-TrFE) gate dielectric layers were prepared by using a mould of PUA fabricated on a PET substrate and crystallized at 140 °C for 4 hours. A bottom-contact top-gate OFET structure (with Ni as the gate electrode, pentacene as the organic semiconductor, and Au as the source/drain electrodes) was used. An organic material of tetratetracontane (TTC) deposited by thermal evaporation at 50 °C and an inorganic material of Al_2_O_3_ was formed by atomic vapour deposition at 200 °C as the organic/inorganic hybrid passivation layer. The as-manufactured P(VDF-TrFE) microstructure was transferred onto the passivation layer by using a thermal tape, prior to depositing a Ni gate electrode. The channel dimensions of the microstructured devices were 40 μm long and 800 μm wide.

### Measurements of SA-mode pressure responses

In order to induce piezoelectricity and pyroelectricity of microstructured P(VDF-TrFE) which responds to pressure and temperature, an on-chip poling process was performed by grounding source (S) and drain (D) electrodes while gate electrode is applied at −100 V.^[36]^ Applying the pressure to the microstructured OFET was conducted by using a cyclic pushing system with a load cell while the device is positioned on a heating block with a temperature controller and a thermocouple. A two-channel arbitrary/function generator (Tektronix AFG 3102) was explored to apply sinusoidal AC V_G_ with a frequency of 0.3125 Hz and amplitude of 20 V. We measured the device characteristics using an HP 4145B semiconductor parameter analyser with time interval of 0.01 s and −20 V of V_D_. The amplitude and mean values of I_D_ induced by AC gate bias were extracted using a fast-Fourier-transform (FFT) method. These processes were also used in stimuli detection from a finger and a blunt stick by positioning the devices on the heating block.

### Realization of detection limit and sensitivity in SA-mode

In order to investigate detection limits and sensitivity of microstructured OFET sensors in SA-mode, a system comprised of a vacuum pump, a tube, and a very light clay, which could apply very low pressure, was prepared. The vacuum pump was connected to a tube to hold and drop the clay for applying low pressure onto devices. We calculated the applied pressure by using the measured weight and surface area of the pieces of the clay.

### Measurements of RA-mode responses to pressure

To measure RA-mode pressure responses of microstructured OFET sensors, a cyclic pushing system with a load cell was used. The system had a function that controlled the pressurizing force and speed as well as the time interval, which enabled measurement of electrical output of microstructured OFETs at different pressures or frequencies. I_D_ of devices was monitored at different pressures ranging from 0.3 to 100 kPa and frequencies ranging from 1 to 10 Hz.

### Measurements and demonstration of sensor arrays

In order to demonstrate the applicability of microstructured OFET sensors, the 4 × 4 arrays manufactured by using the same process for single devices were fabricated. To demonstrate SA-mode pressure detection, a small button was used due to its light weight which can lead to very low pressure on sensor devices. The light button, heated on the hot plate, is positioned on our devices, which enabled us to apply P and T simultaneously, while measuring I_D_ of the microstructured OFET sensors. To demonstrate RA-mode detection, I_D_ change in an array was measured during strongly blowing breath onto the devices. An array is also used for monitoring human body temperature distribution. To compare the temperature distribution of the human skin measured by the sensor array, thermal images of skin temperature distribution were also measured by an IR camera (JENOPTIK Varioscan 3021-ST IR, Germany) after calibration from the thermocouple embedded in a heated metal block that also used for calibration of the sensors and for the SA-mode bimodal temperature detection experiments.

## Additional Information

**How to cite this article**: Kim, D.-I. *et al*. A Sensor Array Using Multi-functional Field-effect Transistors with Ultrahigh Sensitivity and Precision for Bio-monitoring. *Sci. Rep*. **5**, 12705; doi: 10.1038/srep12705 (2015).

## Supplementary Material

Supplementary Information
