# Supplementary material for: A Sensor Array Using Multi-functional Field-effect Transistors with Ultrahigh Sensitivity and Precision for Bio-monitoring
Source: Sci Rep. 2015 Jul 30;5:12705. doi: 10.1038/srep12705 (PMC4520005; doi:10.1038/srep12705)
Supplement: Supplementary Information [file srep12705-s1.pdf]

## SUPPORTING INFORMATION PARAGRAPH

# A Sensor Array Using Multi-functional Field-effect Transistors with Ultrahigh Sensitivity and Precision for Bio-monitoring

*Do-Il Kim[1], Tran Quang Trung[1], Byeong-Ung Hwang[1], Jin-Su Kim[1], Sanghun Jeon[3], Jihyun Bae[4], Jong-Jin Park[5] & Nae-Eung Lee[1,2*],*

[1]School of Advanced Materials Science & Engineering, Sungkyunkwan University, Suwon, Kyunggi-do 440-746, Republic of Korea.

[2]SKKU Advanced Institute of Nano Technology (SAINT), Sungkyunkwan University, Suwon, Kyunggi-do 440-746, Republic of Korea

[3]Department of Applied Physics, Korea University, Sejongro 2511, Sejong 339-700, Korea

[4]Samsung Advanced Institute of Technology, Samsung Electronics Corporation, Yongin, Kyunggi-do 446-712, Republic of Korea

[5]School of Polymer Science & Engineering, Chonnam National University, Gwangju 500-757, Korea

**E-mail: nelee@skku.edu*

*Supporting Figure S1:*

The process flow for manufacturing a microstructured FET sensor

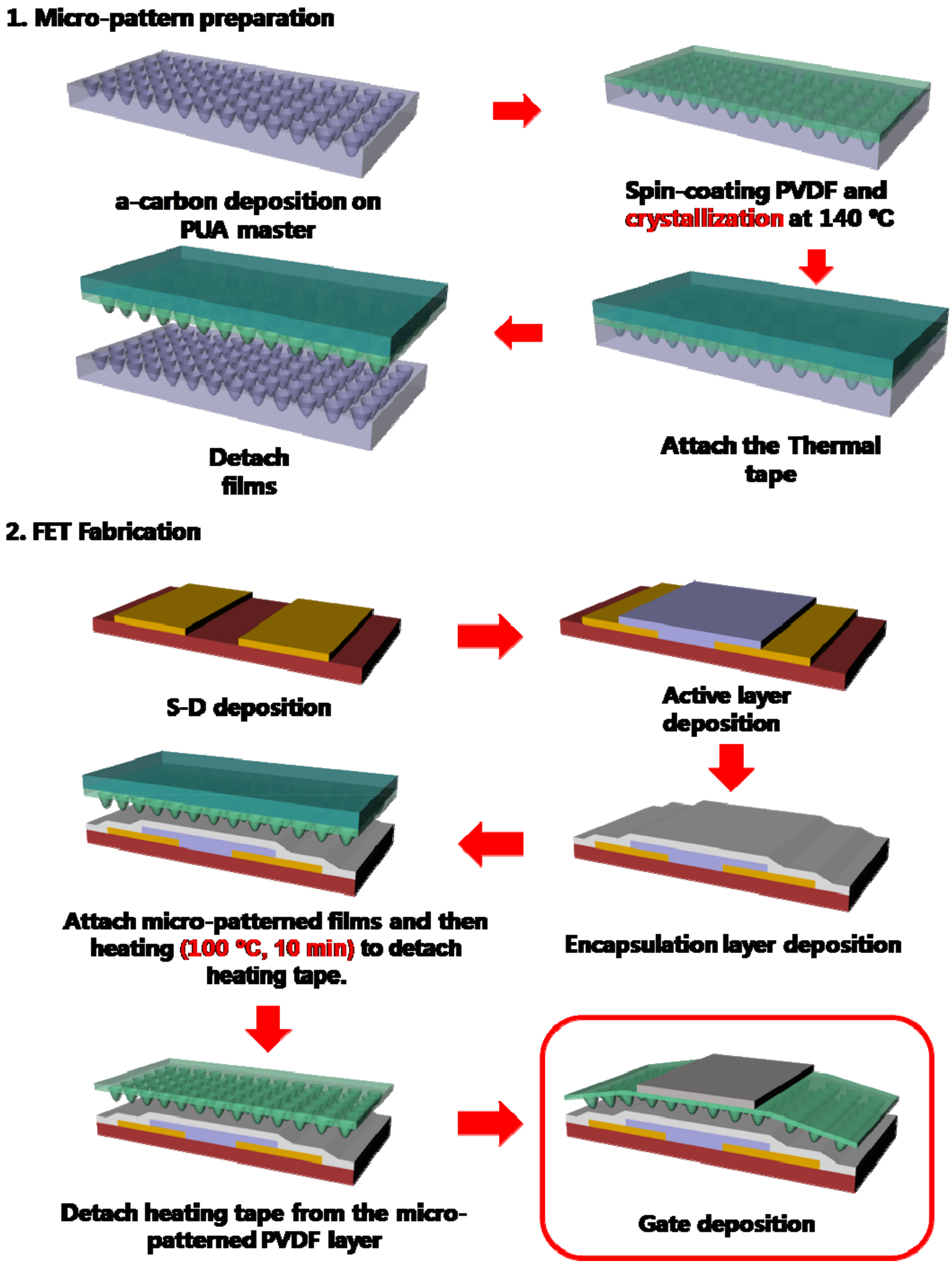

*__Supporting Figure S2:__*

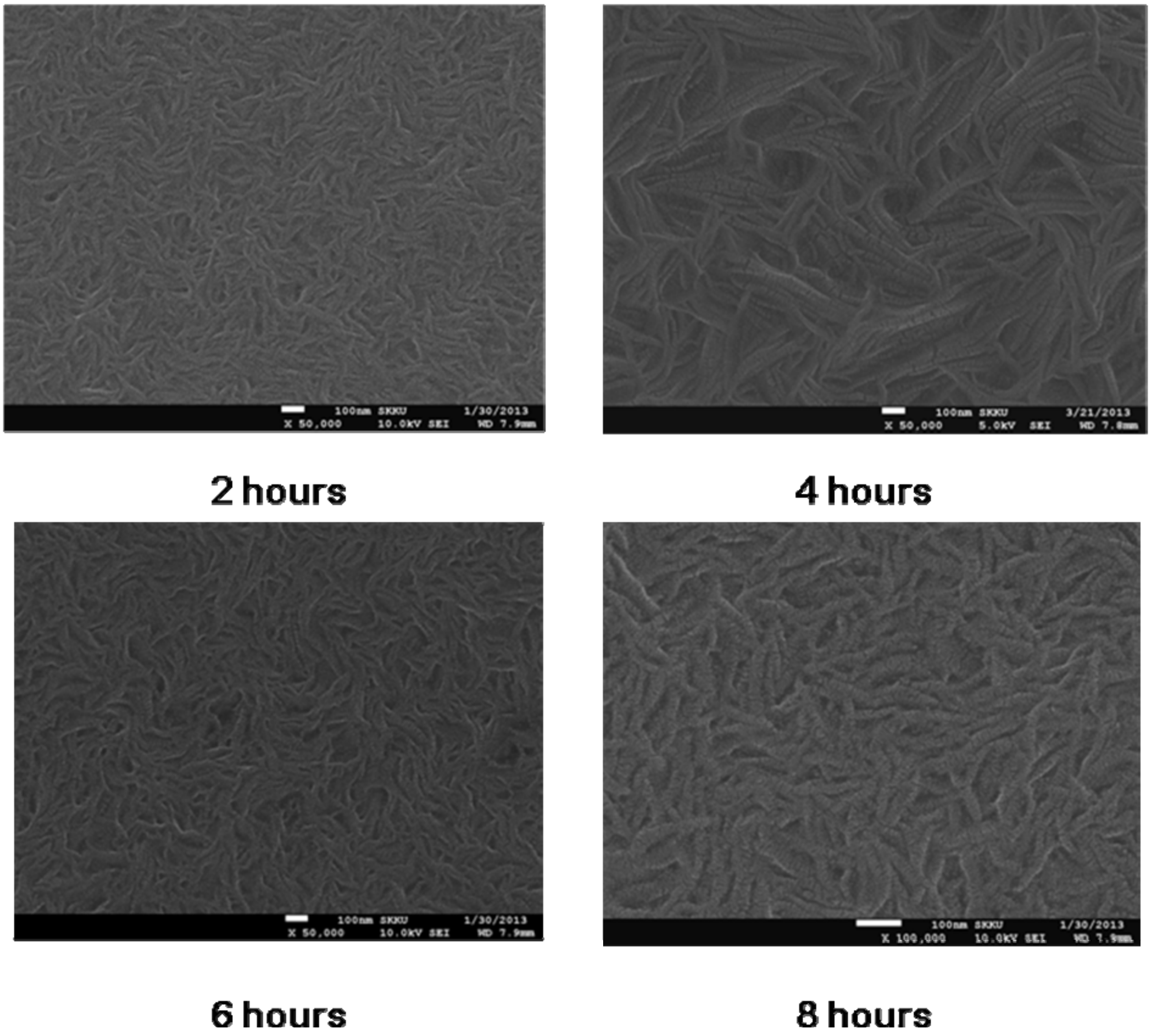


FE-SEM images of thick P(VDF_TrFE) microstructures formed at 140 °C with varying annealing time.

*Supporting Figure S3:*

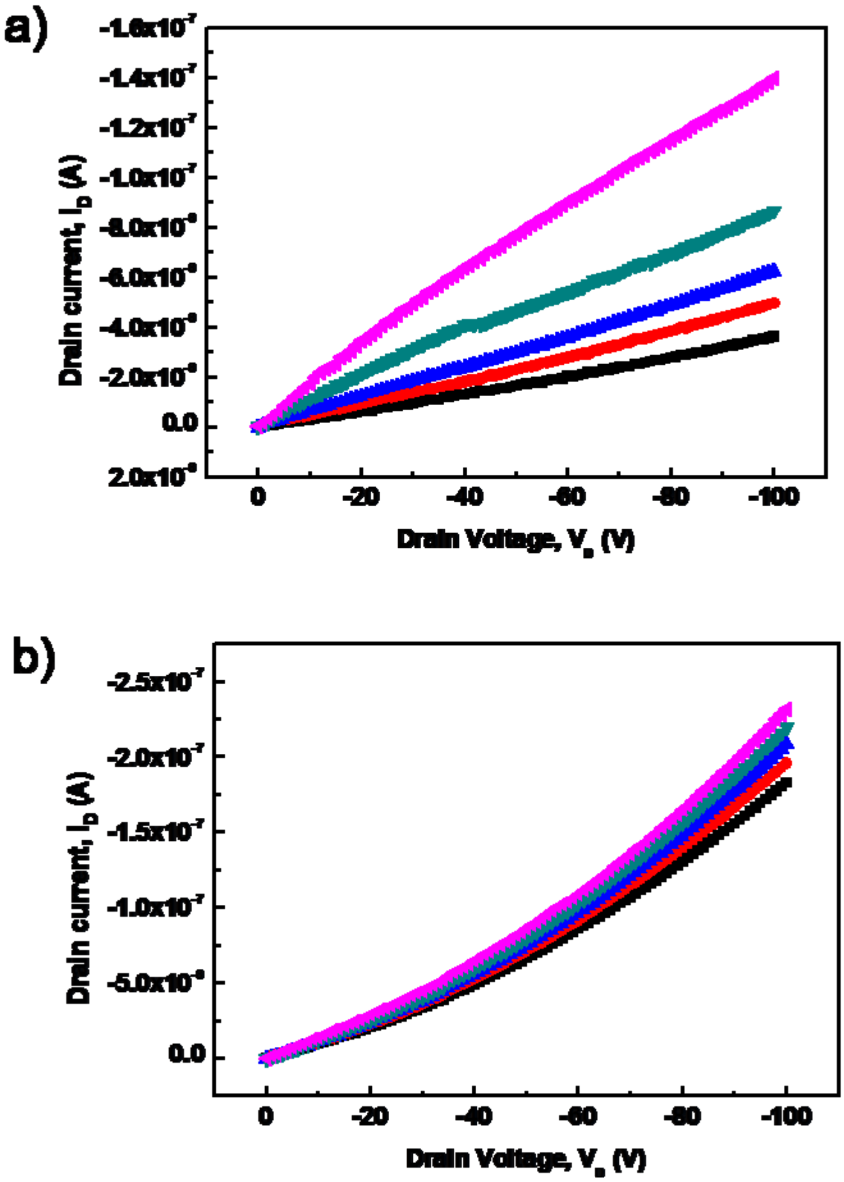


Output characteristics of a microstructured FET sensor (a) before poling and (b) after poling by being biased $V_G$ of -100 V

*Supporting Figure S4:*

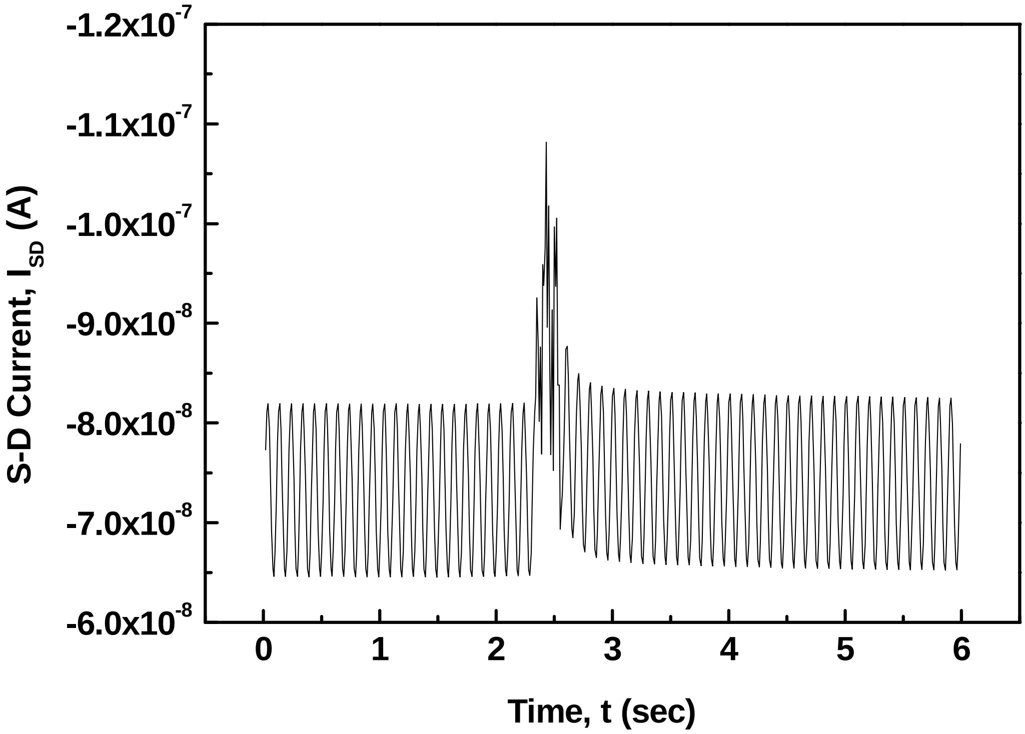


Measured $I_D$ while being biased by AC gate voltage of -20 V at frequency of 10 Hz with applying pressure of 0.3 kPa.

*Supporting Table S1:*

| | $M_1$ [pS/kPa] | $M_2$ [pS/K] | $M_3$ [V/MPa] | $M_4$ [V/K] |
|---|---|---|---|---|
| **P(VDF-TrFE) thin film** | **2.9 ± 0.03** | **345.2 ± 2.6** | **5.02 ± 0.042** | **-2.27 ± 0.082** |
| **P(VDF-TrFE) microstructure** | **44.2 ± 0.28** | **342.1 ± 3.4** | **14.07 ± 0.055** | **-2.06 ± 0.043** |

Extracted M values of FETs using P(VDF_TrFE) microstructure and thin film. $M_1$, $M_2$ : Piezo-resistive and thermal-resistive coefficient of pentacene organic semiconductors respectively, $M_3$, $M_4$ : Piezoelectric and pyroelectric coefficient of gate dielectrics respectively.

*Supporting Table S2:*

| Subject order | Sex | age | Thumb temperature outdoors (°C) | Thumb temperature indoors (°C) |
|---|---|---|---|---|
| 1 | Male | 27 | 20.2 | 35.6 |
| 2 | Male | 27 | 27.0 | 34.3 |
| 3 | Female | 26 | 23.0 | 34.2 |
| 4 | Male | 24 | 13.7 | 33.0 |
| 5 | Male | 28 | 19.5 | 35.0 |
| 6 | Male | 26 | 23.7 | 35.6 |
| 7 | Male | 27 | 23.8 | 33.3 |
| 8 | Male | 25 | 18.8 | 35.4 |

Extracted temperature values of subject's thumb from IR images measured by an IR camera indoors and outdoors (indoor temperature: 24°C, outdoor temperature: 3°C). Average values of thumb's temperatures indoors and outdoors were calculated as 21.2°C and 34.6°C respectively.

## *Supporting Table S3:*

| | Outdoors | indoors |
|---|---|---|
| 1 | 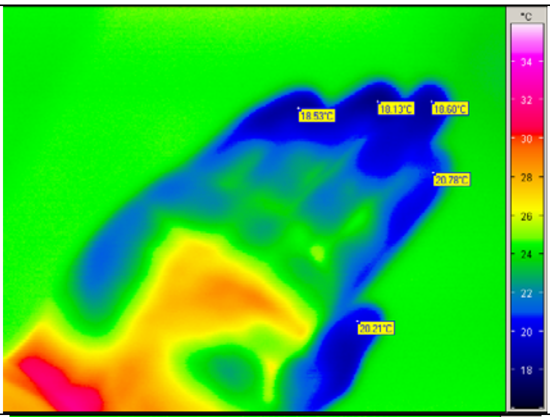  | 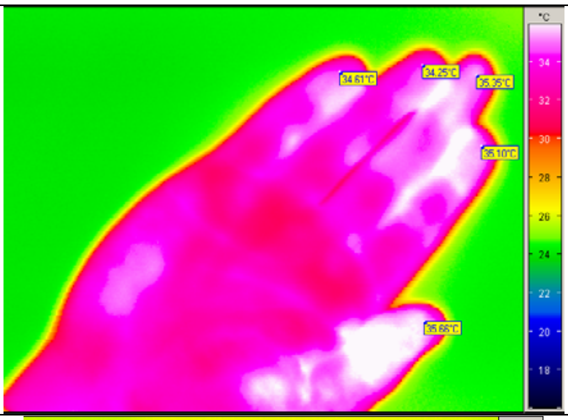  |
| 2 | 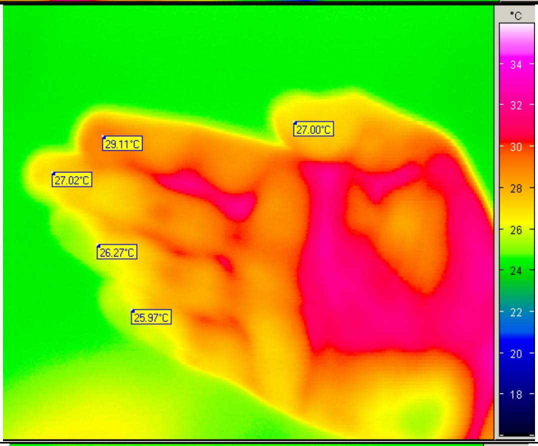  | 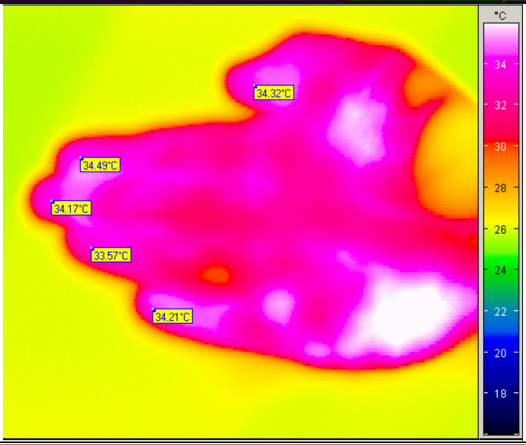  |
| 3 | 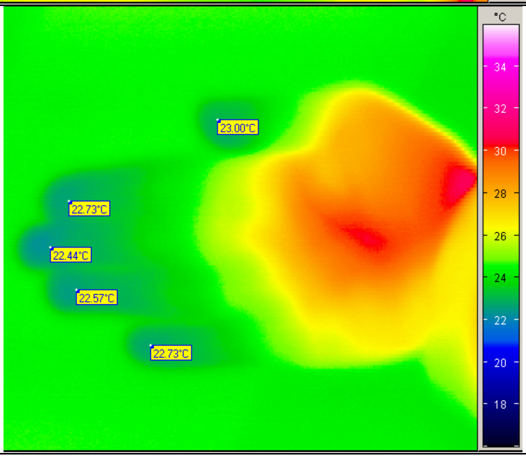  | 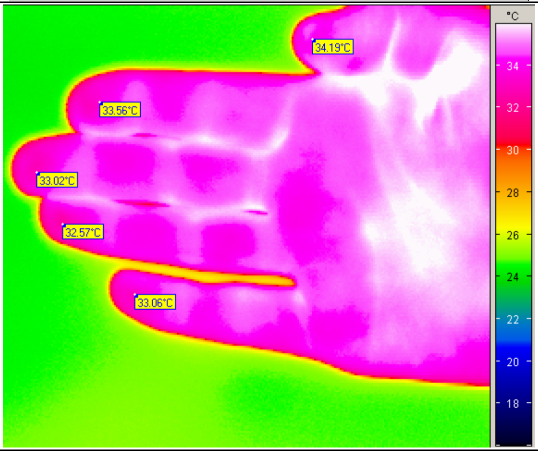  |

**4**

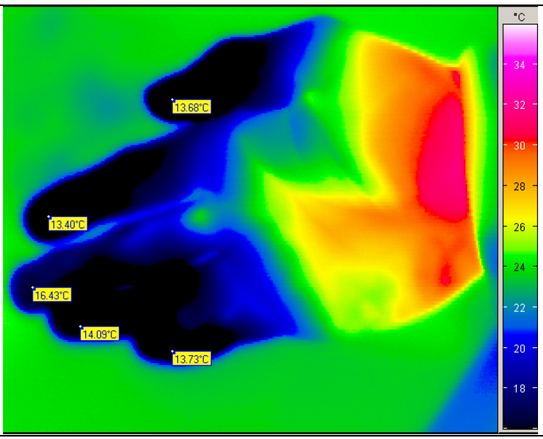
13.68°C
13.40°C
16.43°C
14.09°C
13.73°C
°C 34 32 30 28 26 24 22 20 18

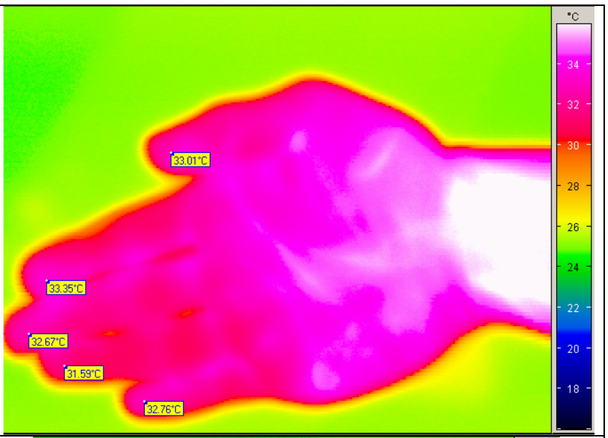
33.01°C
33.35°C
32.67°C
31.59°C
32.76°C
°C 34 32 30 28 26 24 22 20 18

**5**

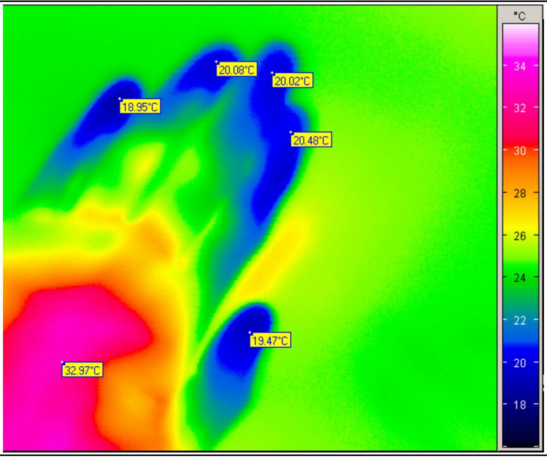
20.08°C
20.02°C
18.95°C
20.48°C
19.47°C
32.97°C
°C 34 32 30 28 26 24 22 20 18

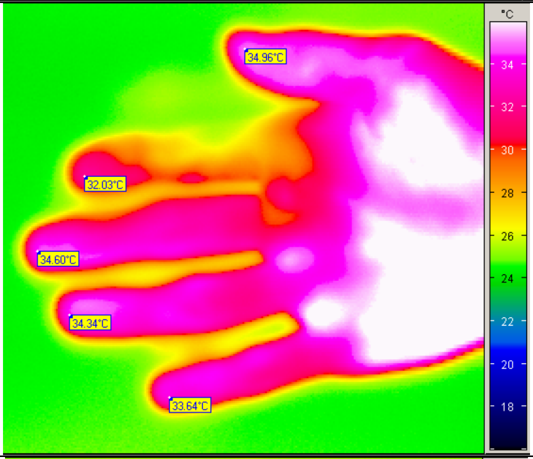
34.96°C
32.03°C
34.60°C
34.34°C
33.64°C
°C 34 32 30 28 26 24 22 20 18

**6**

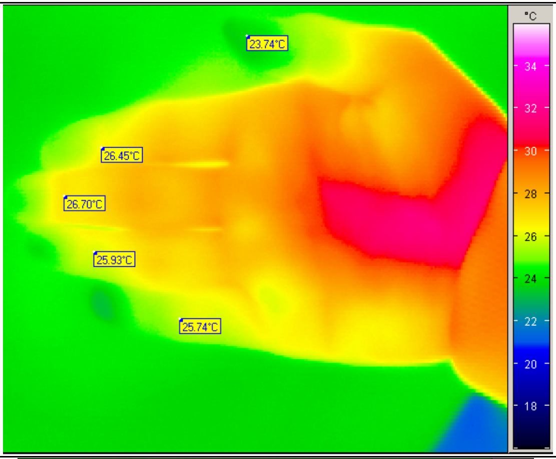
23.74°C
26.45°C
26.70°C
25.93°C
25.74°C
°C 34 32 30 28 26 24 22 20 18

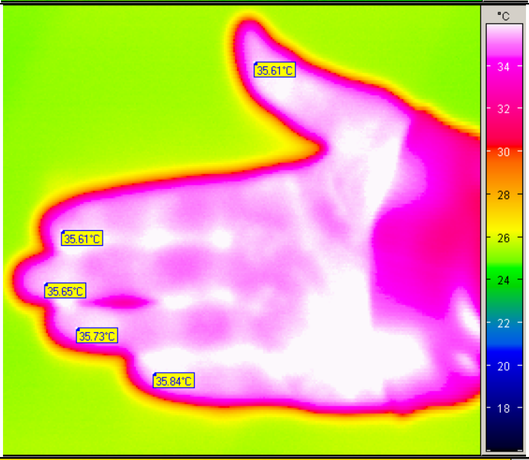
35.61°C
35.61°C
35.65°C
35.73°C
35.84°C
°C 34 32 30 28 26 24 22 20 18

**7**

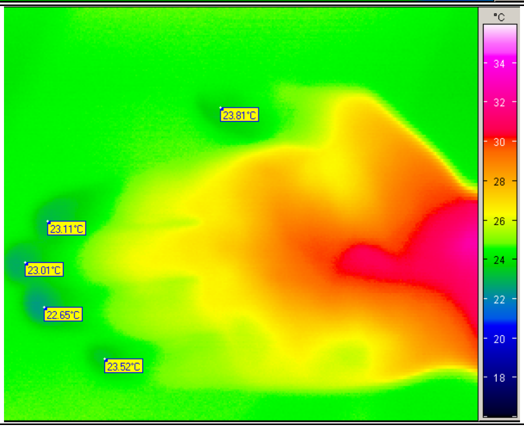
23.81°C
23.11°C
23.01°C
22.65°C
23.52°C
°C 34 32 30 28 26 24 22 20 18

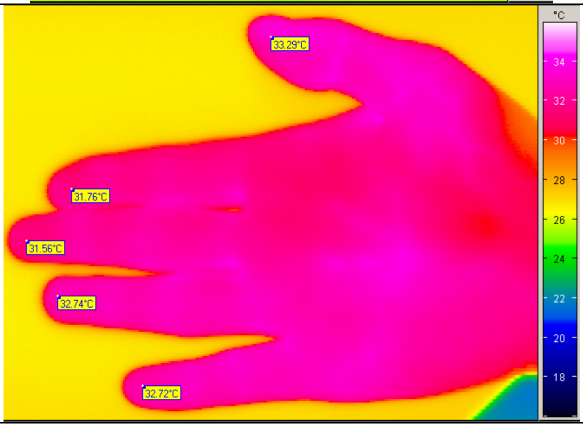
33.29°C
31.76°C
31.56°C
32.74°C
32.72°C
°C 34 32 30 28 26 24 22 20 18

| | | |
|---|---|---|
| **8** | 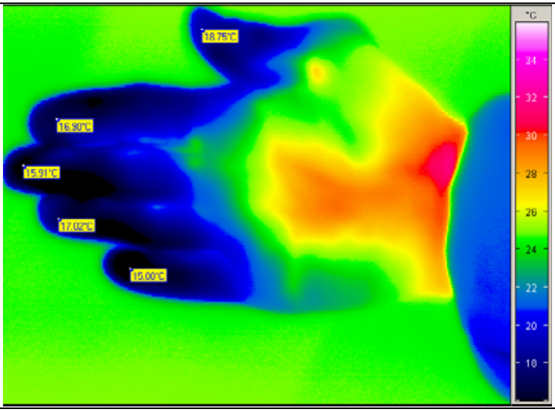  | 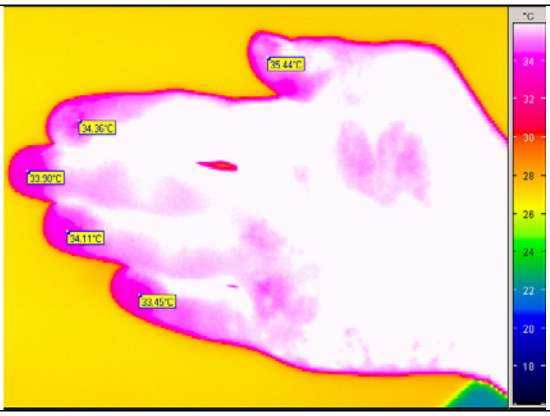  |

IR images of subject's palm measured by an IR camera indoors and outdoors after calibration using a thermocouple in the heated metal block.

*Supporting Figure S5:*

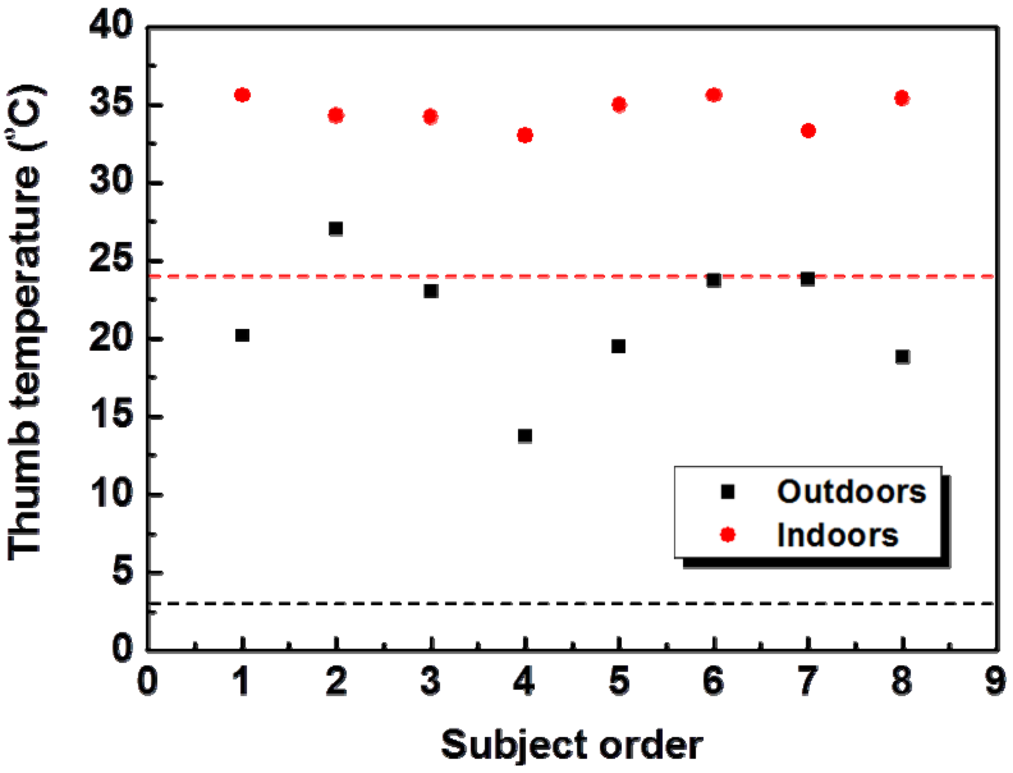


Extracted temperatures of the subject's thumbs from IR images measured by an IR camera indoors and outdoors (red and black dotted lines indicate temperatures measured indoors and outdoors, respectively).

*Supporting Figure S6:*

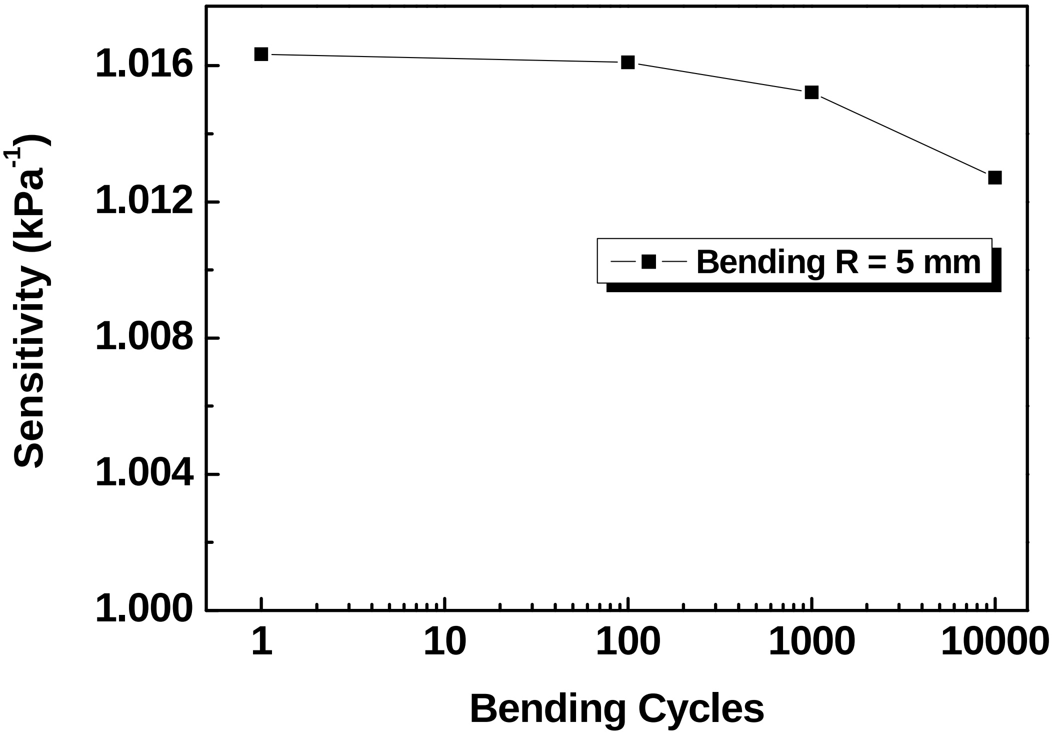


Evaluated sensitivity of microstructure OFET sensors with increasing bending cycles at the bending radius of 5 mm.

*Supporting Figure S7:*

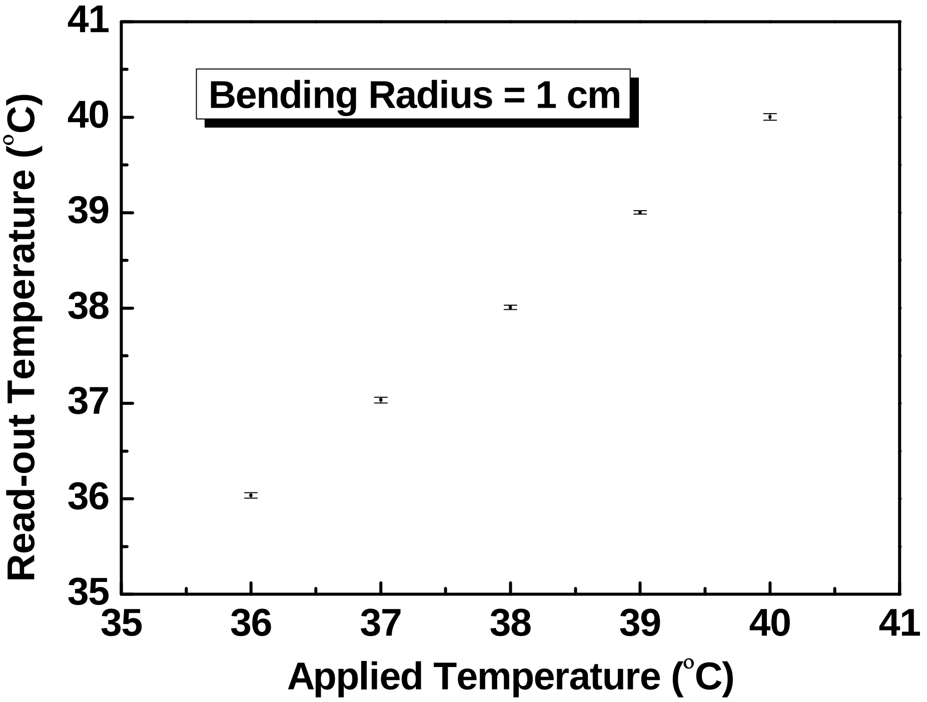


Extracted read-out temperatures under the bent state at bending radius of 1 cm. Error bars indicate the standard deviation of this temperature measurement.
